# The Interventricular Septum Is Biomechanically Distinct from the Ventricular Free Walls

**DOI:** 10.3390/bioengineering8120216

**Published:** 2021-12-15

**Authors:** Michael Nguyen-Truong, Wenqiang Liu, Courtney Doherty, Kristen LeBar, Kevin M. Labus, Christian M. Puttlitz, Jeremiah Easley, Eric Monnet, Adam Chicco, Zhijie Wang

**Affiliations:** 1 School of Biomedical Engineering, Colorado State University, Fort Collins, CO 80523, USA; wenqiang.liu@colostate.edu (W.L.); cdoher@rams.colostate.edu (C.D.); christian.puttlitz@colostate.edu (C.M.P.); 2 Department of Mechanical Engineering, Colorado State University, Fort Collins, CO 80523, USA; kristen.lebar@colostate.edu (K.L.); kevin.labus@colostate.edu (K.M.L.); 3 Department of Clinical Sciences, Veterinary Teaching Hospital, Colorado State University, Fort Collins, CO 80523, USA; jeremiah.easley@colostate.edu (J.E.); eric.monnet@colostate.edu (E.M.); 4 Department of Biomedical Sciences, Colorado State University, Fort Collins, CO 80523, USA; adam.chicco@colostate.edu

**Keywords:** ovine, transmural difference, Fung type model, compliance, septal

## Abstract

The interventricular septum contributes to the pumping function of both ventricles. However, unlike the ventricular wall, its mechanical behavior remains largely unknown. To fill the knowledge gap, this study aims to characterize the biaxial and transmural variation of the mechanical properties of the septum and compare it to the free walls of the left and right ventricles (LV/RV). Fresh hearts were obtained from healthy, adult sheep. The septal wall was sliced along the mid-line into two septal sides and compared to the epicardial layers of the LV- and RV-free walls. Biaxial tensile mechanical tests and constitutive modeling were performed to obtain the passive mechanical properties of the LV- and RV-side of the septum and ventricular walls. We found that both sides of the septum were significantly softer than the respective ventricular walls, and that the septum presented significantly less collagen than the ventricular walls. At low strains, we observed the symmetric distribution of the fiber orientations and a similar anisotropic behavior between the LV-side and RV-side of the septum, with a stiffer material property in the longitudinal direction, rather than the circumferential direction. At high strains, both sides showed isotropic behavior. Both septal sides had similar intrinsic elasticity, as evidenced by experimental data and constitutive modeling. These new findings offer important knowledge of the biomechanics of the septum wall, which may deepen the understanding of heart physiology.

## 1. Introduction

Heart failure is the leading cause of death worldwide [1]. Among the many types of heart failure, left and right ventricular (LV/RV) failure are the most common causes. The investigation of ventricular dysfunction has mostly focused on the adaptation and remodeling of the ventricular free wall, whereas the interventricular septum, the dividing wall between the left and right ventricles, has received much less attention. Therefore, an improved understanding of the septum, including its biomechanical behavior, is needed.

The septum has been traditionally viewed as a single entity and a part of the LV chamber (e.g., the septal thickness is used as an indicator of LV mass), having a similar contribution to LV function as the LV free wall [2]. However, the septum has also been shown to play an important role in RV function. Evidence suggests that RV function was maintained despite elimination or alteration of the RV free wall [3,4,5], and that the induction of septum dysfunction resulted in reduced RV function [6,7]. An excellent review of the septum’s contribution to RV function was provided by Buckberg et al. in 2014 [8]. Moreover, it has been widely observed that in RV failure, secondary to pulmonary hypertension, the septal wall position is shifted towards the left side of the heart with intact LV function [9,10]. Therefore, the septum does not belong to any single chamber; instead, it participates in the physiological function of both ventricles. 

Furthermore, in vivo evidence suggests that the septum wall is a layered structure with transmural variations. For instance, Boettler et al. reported from echocardiography an abrupt change in echogenicity (acoustic reflectance) down the middle of the septum, suggesting a shift in the tissue density dividing the left and right sides. In the left side of the septum, the longitudinal strain and strain rate were larger than those of the radial direction, and the opposite was observed in the right side of the septum [11]. Their findings suggest that the septum wall may be structurally and mechanically different between the LV- and RV-side. Moreover, Holland et al. observed different ultrasonic properties that are sensitive to the tissue structure and function between the two septal sides [12]. In another study, Lindqvist et al. virtually ‘divided’ the septum into three layers with equal thickness and then observed from Doppler echocardiography that the two endocardial layers and the middle layer showed a different strain, strain rate, and tissue anisotropy [13]. Although these imaging studies arbitrarily ‘separated’ the septum into two or three layers without known anatomic boundaries, their results indeed imply a transmural variation of the mechanical behavior within the septum. 

Nevertheless, the full ex vivo characterization of the transmural biomechanical variation of the septum is lacking. To date, only two studies reported the ex vivo mechanical properties of the septum from canine or human samples. These studies tested either the mid-layer [14] or non-specified layer [15] of the septal wall. They found similar elasticity (by constitutive modeling) or maximal stress values between the septum wall and the LV free wall [14,15], with an underlying assumption that there is no transmural difference in the septum. Therefore, the biaxial and transmural variation of the passive mechanics of the septum remains a knowledge gap.

The aims of this study are to characterize the biaxial and transmural variation of the passive mechanical properties of the septum and compare them to their respective ventricle free walls in healthy adult ovine. Sheep were chosen because of the similarities between human and ovine cardiovascular anatomy, physiology, and function [16,17]. Because the septum is formed by ‘fusion’ of the bilateral tubes from two embryonic ventricles that have distinct embryological origins [18,19,20], we chose to investigate the transmural variation by dividing the wall into two sides. We hypothesize that in healthy adults, there are transmural differences between the LV-side and RV-side of the septum, and that these two sides are distinct from that of the LV and RV free walls, respectively. Using biaxial tensile mechanical tests and constitutive modeling, we found that both sides of the septum were significantly softer than their respective ventricular free wall counterparts. Additionally, we found that the collagen content in the septum was significantly less than in the ventricles. At low strains, the septum presented anisotropy in both sides, with a stiffer material property in the longitudinal direction. At high strains, both sides of the septum were isotropic. These biomechanical findings will fill a fundamental knowledge gap in cardiac biomechanics and facilitate a more complete understanding of heart physiology. 

## 2. Materials and Methods

### 2.1. Sample Preparation

**Figure 1:**
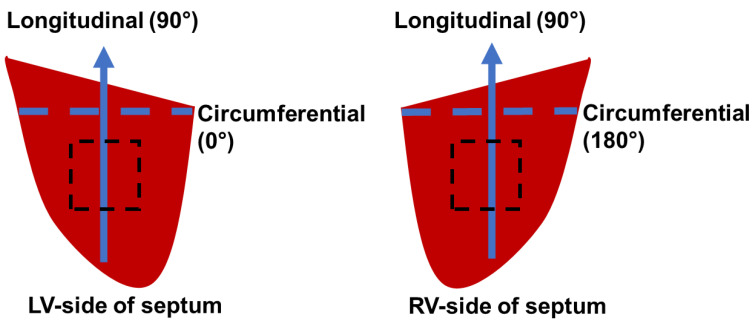
Schematic of orientations for each side of the septum. The arrowhead (longitudinal direction) represents the apex-to-base direction. The black, dashed line square represents the region at which samples were taken for fiber angle measurements and mechanical testing.

Fresh hearts were obtained from adult female sheep (36+ months old: *n* = 12) with no known cardiovascular diseases from unrelated studies at the Colorado State University Veterinary Teaching Hospital. Within 30 h after harvest, the hearts were placed into cardioplegic solution (CPS) (comprised of NaCl, NaHCO_3_, KCl, MgCl_2_, CaCl_2_ in double-distilled water; Alfa Aesar, Ward Hill, MA, USA; Sigma-Aldrich, St. Louis, MO, USA) on ice or at 4 °C to maintain tissue viability. The epicardial layer was extracted from the LV and RV free walls. Next, the septal wall was cleaned with the removal of the trabeculae and papillary muscles, and then sliced approximately down the middle into two halves to elucidate transmural differences between the LV and RV sides of the septum. The ventricle and septal sections were then cut into square sections (septum: 900 mm^2^, ventricle: 625 mm^2^) with a thickness of ~3 mm and kept in CPS and 30 mM of 2,3-butanedione 2-monoxime (BDM) (Alfa Aesar, Ward Hill, MA, USA) at body temperature (37 °C) for at least 10 min to prepare for the passive mechanical tests on physiological conditions. The long axis (apex-to-base) was defined as the longitudinal direction (90°) and the perpendicular axis was defined as the circumferential direction (0° for LV-side or 180° for RV-side) in the septum. The outflow tracts were defined as the longitudinal direction in the ventricular free wall samples [21,22]. A protractor was used to measure the dominant myofiber angle in the septum samples (*n* = 9) (Figure 1). 

### 2.2. Biaxial Testing

After mounting on an in-house biaxial tester, the samples were preloaded with approximately 0.1 N and underwent 10–15 cycles of equibiaxial stretch for preconditioning. An additional 5 cycles of equibiaxial stretch at a quasi-static testing frequency (ventricle: 0.01 Hz and septum: 0.02 Hz) were performed and the data from the last cycle were used for the mechanical analysis. All the preconditioning and tests were performed in a tissue bath of CPS and 30 mM of BDM at 26–37 °C to ensure that the cardiomyocytes were relaxed, and passive biomechanical behavior was obtained [23,24]. All samples underwent a maximum displacement of 20% of the reference configuration to cover the physiological septal strain range [25,26]. Deformations of graphite powder-speckled samples were tracked with a camera (Nikon, Tokyo, Japan). Biaxial stretch forces were obtained by 50-lb load cells (Honeywell Sensotec, Columbus, OH, USA).

The Second Piola-Kirchhoff (P-K) stress—Green strain curves were then derived from the loading curve of the last testing cycle. The formulas were as follows: Second P-K stress (***S***) (S=P/λ, and $P=F/A_{0}$, where *F* is the measured force, *P* is the engineering stress, *A*_0_ is the initial cross-section area, and λ is stretch) and Green strain ($E=\frac{1}{2}\left(\lambda^{2}-1\right)$). The elastic moduli (*M*) were derived as the slope of the stress–strain curve in the low and high strain ranges (i.e., using the first and last 20% of data points of the curve, respectively) [22,27].

### 2.3. Constitutive Modeling 

From our experimental data, we calculated the shear deformation and presented the average normal strains and shear strains in Table 1. Because a majority of the samples had much smaller shear strains than the corresponding normal strains, we neglected shear strain in the constitutive modeling below. The Fung type model uses individual fitting results to predict an average equibiaxial behavior as well as the overall elastic and anisotropic properties of the tissues from the same group. Thus, we adopted this constitutive model in our study.

Green strain tensor (***E***) components were calculated by using the following equations:

(1) $$E_{L}=\frac{1}{2}\left(\lambda_{L}{}^{2}-1\right),\;E_{C}=\frac{1}{2}\left(\lambda_{C}{}^{2}-1\right)$$

where $E_{L}$ and $E_{C}$ are the Green strains in the longitudinal and circumferential directions, respectively; and $\lambda_{L}$ and $\lambda_{C}$ are the stretches in the longitudinal and circumferential directions, respectively.

As in previous studies [28,29], a four-parameter Fung exponential strain energy function (Ψ) was applied:

(2) $$\Psi =\frac{B}{2}\left(e^{Q}-1\right),\;Q=b_{L}E_{L}{}^{2}+2b_{\mathrm{LC}}E_{L}E_{C}+b_{C}E_{C}{}^{2}$$

where $b_{L}$, $b_{\mathrm{LC}}$, $b_{C}$ and *B* are the material constants.

The Second P-K stress and Cauchy stress (**σ**) for an incompressible tissue were calculated using the following equation:

(3) $$S=2\;\frac{\partial \Psi }{\partial C}-pC^{-1},\;\sigma =FSF^{T}$$

where ***F*** is the gradient tensor, **C** is the right Cauchy-Green tensor, and *p* is an unknown hydrostatic pressure to enforce det(**C**) = 1.

Then, the stress-strain relationships in the longitudinal and circumferential directions were derived as:

$$\sigma_{L}=\left(2E_{L}+1\right)\left(b_{L}E_{L}+b_{\mathrm{LC}}E_{C}\right)Be^{\left(b_{L}E^{2}{}_{L}+2b_{\mathrm{LC}}E_{L}E_{C}+b_{C}E^{2}{}_{C}\right)}$$

(4) $$\sigma_{C}=\left(2E_{C}+1\right)\left(b_{\mathrm{LC}}E_{L}+b_{C}E_{C}\right)Be^{\left(b_{L}E^{2}{}_{L}+2b_{\mathrm{LC}}E_{L}E_{C}+b_{C}E^{2}{}_{C}\right)}$$

The Fung strain energy function was fitted to the experimental data for each specimen. The fitting was performed with a Levenberg-Marquardt least-squares algorithm with a tolerance of 10^−8^ in MATLAB. Every fit was checked with at least twenty different initial guesses and all optimizations converged to the consistent values, which indicated the optimization algorithm was independent of the initial guesses. The root mean square (RMS) was calculated to assess the fitting results.

In order to further quantify tissue anisotropy, the stress–strain curves were numerically converted to the equibiaxial stretch condition, where $E_{L}=E_{C}=E$, and then the material difference between the two directions was assessed by an anisotropic parameter *K*:

(5) $$K=\frac{2\left(b_{L}-b_{C}\right)}{\left(b_{L}+b_{C}+2b_{\mathrm{LC}}\right)}$$

Therefore, a positive *K* value ($b_{L}\;>\;b_{C}$) indicated that the material is stiffer in the longitudinal direction, whereas a negative *K* value ($b_{L}\;<\;b_{C}$) indicated that the material is stiffer in the circumferential direction.

By using the converted equibiaxial stress–strain curves, the elasticity at zero load in the longitudinal and circumferential directions were:

$$M_{0,L}=\frac{d\sigma_{L}{}^{equi}}{dE}{|}_{E=0}$$

(6) $$M_{0,C}=\frac{d\sigma_{C}{}^{equi}}{dE}{|}_{E=0}$$

where the $M_{0,L}$ and $M_{0,C}$ are the zero-load elastic moduli derived from the four-parameter Fung type model under the equibiaxial condition in the longitudinal and circumferential directions, respectively.

### 2.4. Histology

Tissue samples were fixed in 10% formalin, dehydrated, embedded in paraffin, sectioned, and stained with Picrosirius Red. Histology images were acquired by an inverted microscope (Motic AE31E, Motic, San Antonio, TX, USA). Three regions of interest to represent the overall tissue were randomly selected. To quantify the collagen content, the areas that were positive for collagen (dark red) were identified using a color thresholding function in ImageJ (NIH, Bethesda, MD, USA), and the collagen area fraction (%) was calculated as the ratio of the collagen area to the total tissue area. All images were analyzed by the same observer, who was blinded to the group information. Since the ventricle and septum tissues were stained in different batches, the non-specific staining in the background was different, and thus different thresholding values were set for these batches. However, this did not affect the identification of the positive staining of collagen fibers, and the images from the same batch were acquired at the same setting.

### 2.5. Statistical Analysis

Data are presented as mean ± SEM. The Mann–Whitney U-test was used for the myofiber angle and mechanical comparisons in the septum. One-way ANOVA and Tukey post-hoc tests were performed between directions, and between the septal and ventricular *M* and collagen data. *p* < 0.05 was considered statistically significant.

## 3. Results 

### 3.1. Transmural Change in Septum Myofiber Orientation

From the myofiber angle measurements, we observed a change in the fiber angle from the LV-side to the RV-side of the septum. As shown in Table 2, the myofiber angle shifted from a longitudinal orientation on the LV-side to a circumferential orientation in the midwall, and then back to a longitudinal orientation on the RV-side of the septum. Therefore, the transmural distribution of myofibers was to some degree symmetric (‘mirrored’) between the two sides of the septum.

### 3.2. Softer Septal Side Compared to the Corresponding Side of Ventricular Free Wall

**Figure 2:**
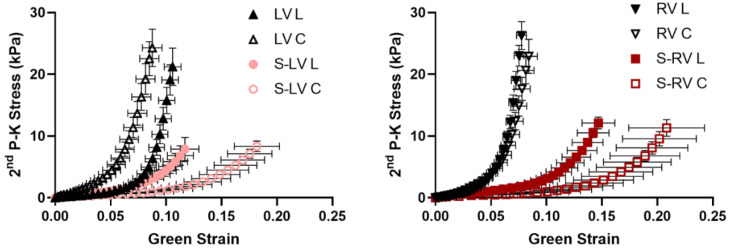
Average stress–strain curves of the ventricular free walls (LV, RV) and the LV-side and RV-side of the septum. *n* = 12 for septum; *n* = 8 for ventricles. S-LV: LV-side of septum; S-RV: RV-side of septum; L: longitudinal direction; C: circumferential direction.

The average stress–strain curves of the ventricular free walls and both sides of the septum are presented in Figure 2. In both sides of the heart chamber, the ventricular free wall showed stiffer behavior compared to the corresponding side of the septum, which is demonstrated by the leftward shifted stress–strain curves. This difference was clearly seen in both the longitudinal and circumferential directions of the respective septum side with its corresponding free wall.

**Figure 3:**
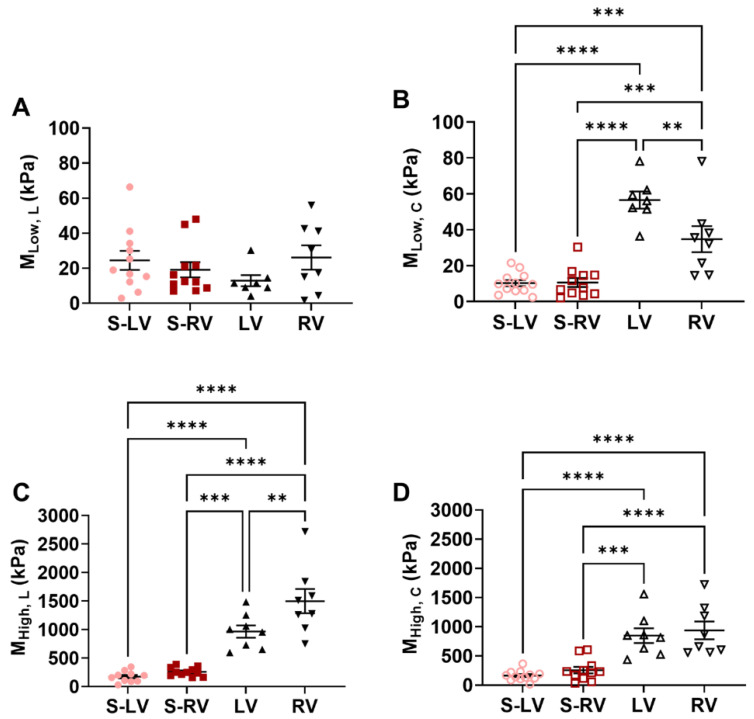
Comparisons of elastic moduli (*M*) at low (**A**,**B**) and high (**C**,**D**) strains from the septal sides and ventricles. Left columns show the *M* obtained in the longitudinal direction and right columns show the *M* obtained in the circumferential direction. ** *p* < 0.01, *** *p* < 0.001, **** *p* < 0.0001. *n* = 10–12 per group for septum; *n* = 7–8 per group for ventricles. S-LV: LV-side of septum; S-RV: RV-side of septum.

Next, we compared the elastic moduli (*M*) between the groups and between the directions at low and high strains (Figure 3). In the low strains, the free walls had larger *M* than the corresponding side of the septum in the circumferential direction only, indicating a stiffer ventricle free wall than septum wall. In the high strains, the *M* was larger in the free walls than the corresponding septum side in both directions, indicating a stiffer ventricle free wall than septum wall at larger deformations. 

### 3.3. Transmural Differences in the Septum Obtained from Experimental Data

**Figure 4:**
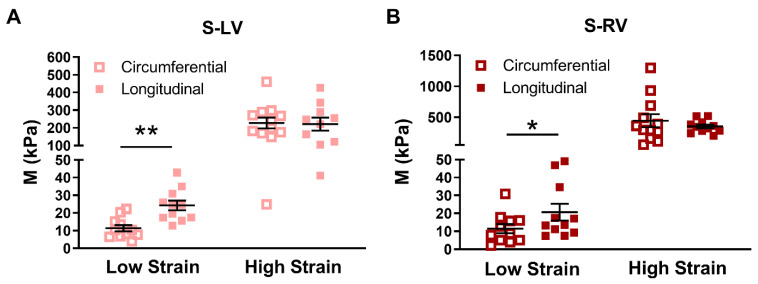
(**A**,**B**) Comparison of elastic moduli (*M*) between biaxial directions in the left and right sides of the septum. Significant anisotropy in both S-LV and S-RV at low strains, and the degree of anisotropy was much stronger in the S-LV. * *p* < 0.05 vs. circumferential in the same strain range. ** *p* < 0.01 vs. circumferential in the same strain range. *n* = 10–12 per group for septum; *n* = 10–12 per group. S-LV: LV-side of septum; S-RV: RV-side of septum.

Similar anisotropies are shown from the low-strain elastic modulus (*M*) between the two sides of the septum (Figure 4A,B). Compared to the *M* in the circumferential direction, the *M* in the longitudinal direction was higher. There was stronger (*p* = 0.002) anisotropy in the LV-side of the septum compared to the RV-side (*p* = 0.049). At high strains, however, both sides of the septum presented isotropic behavior. Next, we compared the elastic moduli (*M*) of the two sides from the same direction. There was no difference in the *M* between the two sides of the septum in all strain ranges or in both directions, although there was a trend of a stiffer RV-side septum compared to the LV-side in the high strain range in the circumferential direction (*p* = 0.06).

### 3.4. Transmural Differences in the Septum Obtained from Computational Modeling 

We fit the septum equibiaxial stress–strain curves using a four-parameter Fung type model described previously in several myocardium studies [28,29]. A good fitting with the experimental data was observed for all the tissues and in both directions (i.e., with low RMS values), and the fitting results are summarized in Table 3. We did not observe any statistical significance in the model parameters (Table 3) between the LV-side and RV-side of the septum, indicating a similar material property (overall elasticity) of the tissue. The simulated stress–strain curves that were numerically converted to the equibiaxial condition showed similar shapes as our experimental data. 

**Figure 5:**
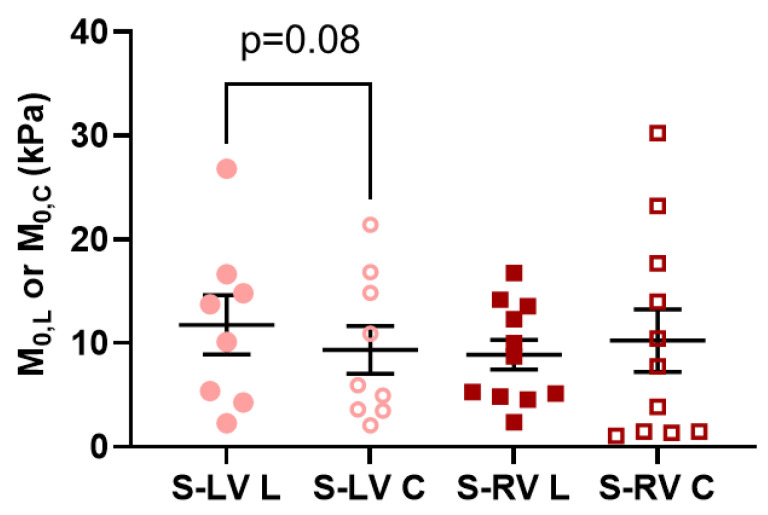
Longitudinal (L) and circumferential (C) zero-load elastic modulus (*M*_0_) at zero load derived from the fitting parameters for each side of septum. *n* = 9–11 per group. S-LV: LV-side of septum; S-RV: RV-side of septum.

We further compared the zero-load elastic modulus (*M*_0_) derived from the model fitting in each ventricle type, and in different directions. This parameter serves as an indicator of myofiber stiffness. As shown in Figure 5, there was a trend of larger $M_{0,L}$ compared to the $M_{0,C}$ in the LV-side septum (*p* = 0.08), indicating that the LV-side was more anisotropic and stiffer in the longitudinal direction than the circumferential direction (Figure 5). There was no difference in the *M*_0_ between directions for the RV-side septum. Moreover, the anisotropic parameter *K* was positive for both sides (0.40 ± 0.17 in LV-side and 0.25 ± 0.28 in RV-side), which indicated a stiffer behavior in the longitudinal direction. These results agreed with our experimental data, especially at the low strain range (Figure 4A,B).

### 3.5. Difference in Collagen Content between Septum and Ventricular Tissues

**Figure 6:**
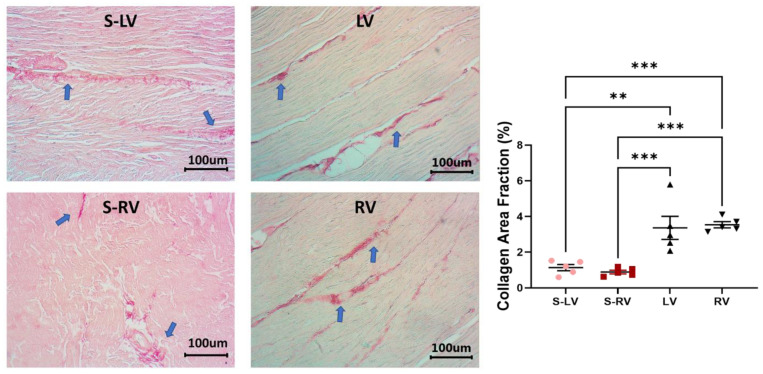
Representative histology images (**left**) and the comparisons of collagen area fraction (**right**) between the S-LV, S-RV, LV, and RV groups. Arrows show the positive staining of collagen fibers. ** *p* < 0.01, *** *p* < 0.001. *n* = 5 per group. S-LV: LV-side of septum; S-RV: RV-side of septum.

When comparing the overall collagen content between both sides of the septum, we found a trend of higher collagen content in the LV-side compared to the RV-side, but the difference was not significant. Furthermore, the collagen content in the LV or RV was significantly higher than that of the corresponding side of the septum (*p* < 0.05), indicating a marked reduction of collagen in the septal wall compared to the ventricular free walls (Figure 6). This may explain the difference in elastic moduli between the ventricle and its respective septum side.

## 4. Discussion

This is the first study to characterize the ex vivo passive mechanical properties of the LV-side and RV-side of the septum and compare them to their respective ventricles in healthy, adult ovine. We found that the ventricular free walls were stiffer than their respective septal counterparts. Histology examination showed that the collagen amount was higher in the ventricles compared to their respective septal counterparts. This suggests that the septum has unique structure–function relationships compared to both ventricles. Transmurally, the two sides of the septum showed a symmetric distribution of fibers that shifted from the longitudinal direction at the endocardial layer to the circumferential direction at the mid-layer. Thus, similar anisotropic behavior existed in both sides (i.e., stiffer longitudinal behavior than circumferential behavior), but the degree of anisotropy was stronger in the LV-side. The intrinsic elasticity was similar between the two sides of the septum, and in both directions. Our results suggest that the septum should not be treated as a part of the LV or RV, and the unique transmural distribution of the biomechanical properties of the septum will assist in the understanding of left and right ventricular function.

### 4.1. Different Mechanical Behavior between Ventricle Free Walls and Septum

The two ventricles have different embryological origins. The early heart ventricles, which are formed from bilateral tubes, fuse to form the septum during cardiac development [18,19,20]. Therefore, it is reasonable to speculate that the transmural differences in mechanical behavior are likely attributable to the different cell lineages [19] and the growth patterns and demands between the two ventricles [9]. To our knowledge, only two studies have performed ex vivo mechanical tests on the septum, and both neglected the transmural variations in the septum. Novak et al. was the first to compare the biaxial mechanical properties of the inner, middle, and outer layers of the LV and middle portion of the septum in canines [14]. They did not find significant differences in the stored strain energy—inferring similar elasticity of the tissues. However, a trend of a stiffer inner and outer LV free wall than the middle layer of the LV and septum was noted. More recently, Sommer et al. obtained peak Cauchy stress in the human LV and RV free walls and septum [15]. They found a higher stress in the RV compared to the LV and septum, which had similar stresses. However, the finding may be compromised by two factors: first, the samples were from patients with cardiac related diseases; second, the measurements of the septum and RV tissues at high stretches were missing due to tissue failure close to the hook, and there were only three RV samples. Therefore, a complete mechanical comparison between the septum and the LV and RV free walls is not available from this study.

Our study is the first to examine the biaxial mechanical behavior of the septum wall in two halves and compare each side of the septum to its respective ventricular free wall. We found significantly stiffer ventricular free walls than the corresponding side of the septum, and the stiffer behavior was more pronounced in the higher stretch (strain) regions. It is known that collagen is a load-bearing protein and that more collagen is correlated with stiffer elastic behavior [30]. The larger collagen content in the ventricles than the septum may thus explain the observation. Other structural differences, such as myofibers, may contribute to the tissue mechanics as well. Unfortunately, there is a lack in understanding of whether the cardiomyocytes in the septum are identical to those in the ventricular free walls.

Our data partly agreed with the previous reports noted above. For example, it is found that the outer and inner layers of the LV free wall tended to be stiffer than the midwall of the septum [14], and the RV free wall showed higher peak stresses compared to the septum for the same strain [15]. Akin to the former findings, we observed similar mechanical behavior between the LV and septum at the low strain range in the longitudinal direction, and a stiffer RV free wall compared to the septum. We did not intend to make in-depth comparisons between our study and previous studies because of the different layers of myocardium used and different biaxial orientations (outflow tract or apex-to-base direction as the longitudinal direction in our study versus the main fiber direction as the longitudinal direction in previous studies). The relatively new biaxial axes system has been adopted by other studies, and it provides more information about the relation of tissue mechanics to ventricle function (see our discussion in Section 4.3). Overall, our finding that the septum is intrinsically more compliant than the ventricles should be studied further in different species and with the use of multi-scale approaches (from organ to cell biomechanics).

The stark differences in the elasticity between the free walls and septum can explain in vivo findings of the different contributions of the septum and free walls to the overall ventricular function. Assuming the similar mechanical properties and contractility of the septum and LV free wall, we would expect 16–35% of the cardiac work to be contributed by the septum (versus 65–84% contribution by the LV free wall), based on its volume fraction from the sheep and human data [31,32]. However, Ostenfeld et al. reported that, in healthy subjects, the septum contraction contributed only ~8% of the stroke volume, while the LV free wall contributed ~96% of the stroke volume [33]. Similar findings were shown by Stephensen et al. where ~7% of the septal motion contributed to the LV stroke volume [34]. Similarly, if we treat the septum as part of the RV chamber, it contributes somewhat similarly as the RV free wall in pumping, where 36–54% of cardiac work is attributed to the septum (versus 45–64% contribution by the RV free wall) [31,32]. Thus, the mechanical differences between the septum and ventricles found in our study suggests that the septal wall is not ‘similar’ to the ventricular free walls, and the mechanical difference may be linked to the altered muscle shortening velocity, leading to a ‘weaker’ contribution to the overall cardiac function than that of the ventricle free walls.

### 4.2. Fiber Orientation in the Septum

It has been reported that the ventricular free walls exhibit a transmural fiber orientation shift [21,35]. In the LV free wall, studies have reported that the myofibers shift from a longitudinal (endocardium) to a circumferential (midwall) to a longitudinal (epicardium) orientation [35,36]. Other measurements of the LV myofiber transmural changes from using myocardial diffusion imaging [37] or a protractor [38] documented a total angle change of 120°–180°. In the RV free wall, the myofiber angle changed from a circumferential to longitudinal orientation in the epicardium to endocardium, respectively [21]. Therefore, it is not surprising that the septum wall presents a transmural change in the myofiber orientation as well.

In the present study, we had a preliminary examination of the transmural change of fiber orientation in the septum, and we originally found that the average fiber orientation shifted from 78 ± 3° (LV-side) to 9 ± 2° (midwall) and, finally, to 108 ± 8° (RV-side). Our data showed ~150° for the main myofiber angle transmural shift through the entire septum. The transmural change may be related to the individual support of the pumping demand for each ventricle. For instance, the longitudinal myofiber orientation on each endocardial side are, potentially, better aids in blood ejection along the outflow tracts of each ventricle.

### 4.3. Anisotropy Behavior of the Septum

As we mentioned above, the ex vivo mechanical properties of the septum reported in prior studies used the main fiber and cross-fiber directions as the testing axes. It is not surprising that these studies reported similar anisotropy, i.e., a stiffer behavior in the fiber direction than cross-fiber direction, probably due to the aligned myosin–actin cross-bridge, and titin in the ‘fiber direction’ (direction of myofiber shortening). However, such measurement cannot provide the anatomic relations between the tissue anisotropy and the ventricular deformation in systole and diastole, thus preventing an investigation of the relation between the biaxial mechanical behavior and the physiological function of the myocardium. Furthermore, using the main fiber and cross-fiber coordinate system for biaxial tests can be problematic for tissues that have a significant transmural change in the fiber orientation. The myocardium tissue (LV/RV free wall) is composed of helical and heterogenous myofibers that vary in orientations transmurally [8,39]; similarly, we observed marked transmural changes in the myofiber orientation in the septum (Table 2). Thus, it is difficult to accurately determine the ‘main fiber’ direction of a myocardial wall in a single section plane.

In all septum tissues, we observed stiffer behavior in the longitudinal direction in the low strain range (*p* < 0.05, Figure 4A,B), indicating a similar pattern of anisotropic behavior of the LV-side and RV-side of the septum. Since it is shown that the low strain mechanical behavior of the myocardium is primarily due to myofibers [22,40], the appearance of tissue anisotropy only in the low strain range indicates that the myofibers are the main cause of septum anisotropy. Furthermore, although the anisotropy is quite ‘mirrored’ between the two sides, the anisotropy in the LV-side of the septum was highly significant (*p* < 0.01), whereas that in the RV-side of the septum was marginal (*p* = 0.049). The different degrees of anisotropy between the two sides have been evidenced by the different in vivo strains (a surrogate of elasticity) between the longitudinal and radial axes of each septum side. Boettler et al. found the global longitudinal and radial strain difference was larger in the septal LV-side than the RV-side (LV-side: Δ strain ~13%; RV-side: Δ strain ~5%), indicating a more anisotropic behavior of the left-side of the septum than the right-side [11]. In the current study, we did not obtain the mechanical property of the septum in the radial (i.e., transmural) direction, but we found a similarly stronger tissue anisotropy in the septal LV-side than the RV-side. This was further confirmed by our modeling results of the LV-side zero-load elastic modulus (*M*_0_), which tended to be larger than that of the RV-side. Given that the two septal sides have different strains and different degrees of anisotropy, it is reasonable to anticipate different elastic resistances in the different axes or septum sides, which results in varied impacts on the LV and RV diastolic functions. Therefore, a further investigation of the relations of septum mechanics and ventricular function is needed. 

### 4.4. Transmural Differences between LV-Side and RV-Side of the Septum

We also compared the strain-dependent elastic moduli and modeling parameters of elasticity between the two sides of the septum. We did not observe any difference in the intrinsic mechanical property. We further examined the potential differences in the myofibers and collagen fibers in the septum sides. There was no difference in the zero-load elastic modulus (*M*_0_) predicted from the constitutive model, which suggests that the myofiber stiffness is comparable between the two sides. The examination of the collagen fraction also showed no significant difference between the two sides. Therefore, the main transmural change in the septum is related to the orientation of the myofibers (anisotropy), not the material properties or tissue composition.

### 4.5. Limitations

In this study, we sliced the septum into approximately two equal halves after cleaning the endocardial surfaces. To our knowledge, there is no anatomic mark to distinguish the LV- and RV-side of the septum, although the two sides share distinct embryologic origins [19,20]. The only data about these thicknesses is provided by Boettler et al. [11], which showed ~1 mm of thickness difference at diastole (with the LV-side to be thicker). However, this difference is about the typical required resolution for medical ultrasound. Thus, due to a lack of clear data to guide the slicing, we tested the two layers with similar thickness. Such methodology may lead to under or overestimation of the transmural differences of the septum. Another limitation is that the same long axis (apex-to-base) was defined as the longitudinal direction of the septum on both sides. However, it is possible that the two sides may have different ‘shortening’ directions, as we defined for the ventricular free wall (outflow tract direction). Future studies should investigate the in vivo shortening axis for each side of the septum. Finally, we assumed a negligible shear deformation, but a certain level of shear deformation occurred (as shown in Table 1). Since it is impossible to completely rule out the shear deformation for anisotropic tissues in biaxial tests, a better characterization of the septum biomechanics should also involve the shear or triaxial test to fully measure the 3D mechanical behavior of the anisotropic tissue.

## 5. Conclusions

In summary, this original study investigates the transmural variation of the ex vivo passive mechanical properties of the septum in healthy adult ovine. The septum sides were significantly softer than their corresponding ventricular free walls, and the collagen content was less than that of the ventricular walls. At low strains, there was similar anisotropic behavior between the two sides, but the degree of anisotropy was stronger in the LV-side than the RV-side. At high strains, both sides were isotropic. Our results suggest that the septum should not be treated as a part of the LV or RV, and that the tissue presents distinct structure–function relationships from the ventricular free wall. The investigation of septum biomechanics will further reveal the biomechanical mechanism of ventricular function and the dysfunction in heart failure progression.

## Figures and Tables

**Table 1:** Maximum normal strains and the corresponding shear strains of the septum samples. ***ε****_L_* and ***ε***_C_ are normal strains in the longitudinal and circumferential directions, and ***ε**_LC_* and ***ε**_CL_* are shear strains in the shear directions. Data presented as mean ± SEM.

Group	*ε_L_*	*ε_C_*	*ε_LC_*	*ε_CL_*
LV-side (*n* = 12)	0.11 ± 0.01	0.18 ± 0.02	0.03 ± 0.02	0.05 ± 0.02
RV-side (*n* = 12)	0.15 ± 0.02	0.21 ± 0.03	0.01 ± 0.02	0.01 ± 0.02

**Table 2:** Dominant myofiber angle of the LV-side, midwall, and RV-side of the septum. Longitudinal direction was defined as 90°. Data presented as mean ± SEM. * *p* < 0.05 vs. LV-side and ^#^ *p* < 0.05 vs. RV-side.

	LV-Side (*n* = 4)	Midwall (*n* = 9)	RV-Side (*n* = 4)
Myofiber angle (°)	78 ± 3	9 ± 2 *^,#^	108 ± 8

**Table 3:** Model fitting results. Data presented as mean ± SEM.

Septum	*b*_L_	*b*_C_	*b*_LC_	*B* (kPa)	RMS (kPa)
LV-side	62.54 ± 12.62	40.84 ± 7.74	0.004 ± 0.002	0.21 ± 0.04	0.21 ± 0.04
RV-side	46.50 ± 7.21	32.66 ± 7.63	0.005 ± 0.001	0.19 ± 0.04	0.30 ± 0.04

$b_{L}$, $b_{C},$ $b_{\mathrm{LC}}$, and *B* are the material constants, and RMS is root mean square. L and C represent longitudinal and circumferential directions, respectively.

## Data Availability

The data presented in this study are available within the article and upon reasonable request.
